# Rev. Mr. Rowland Hill, on Vaccination

**Published:** 1807-02

**Authors:** Rowland Hill

**Affiliations:** Surry Chapel


### *To the Editors of the Medical and Physical Journal.*

GENTLEMEN,

I Felt myself exceedingly gratified at the notice you took of my little Treatise in defence of Vaccination, which appeared before the public a few months ago.

Not being myself possessed of any medical or surgical knowledge, I had not the least claim upon your attention. As it respects vaccination, however, I have acquired all the information in my power, collected not only from writers of the first ability, belonging to the profession, but have exercised a considerable degree of patience, by reading all that was necessary from its few opponents. And to attend to pamphlets like theirs, must be patience indeed, as most of their little party are pretty correct in following the language of Doctor Moseley, their leader, who has been well described as being possessed of a most masterly knack of conjoining "the ravings of Bedlam with the tropes of Billingsgate;"* and I verily believe, if that *gentleman* was to be shut up in his chamber till he could think with candour, write with modesty, and be ruled by reason, he would be a prisoner for life. In one page of his last publication, when his ravings were at the highest

* See Edinburgh Review, No. xvii, p. 38.

highest pitch, he lays himself open to the severe corrections of the law. I mention a young woman, not much beyond childhood, who frequently vaccinates herself, and without the least effect, as a strong and irrefragable proof of the perfect innocency of the vaccine fluid on the human constitution. Now, this most strange creature, according to his rhapsodical and ranting style of writing, insinuates, that she is six years advanced beyond the age I mentioned in my publication; and then more than insinuates, that she positively is my w——.

Now, nothing screens this man from the castigations he deserves for his libel on my character, but this one consideration; it is impossible for such a writer to do harm to any person's character but his own.

I am ashamed to present before you a single quotation or reference further from such a publication;* but as it rests upon this very serious and important consideration, the excessive mischiefs and destructive consequences produced among the lower orders of people thereby; and of this there is terrible evidence from day to day. Let a thinking man take his station in any populous abode; let him count a hundred people as they pass by; let these afterwards be collected together. With these we will suppose he enters into close argument and solid conversation; how many of these will he discover to be men of real intelligence, deliberate judgment, and correct determination? Shall I say one in ten? Considering human weakness, and a general want of a proper education to improve the mind, I fear even this is a greater average than can be allowed.

A famous Doctor, who was accustomed to retail his quackery in this neighbourhood, and who, by the bye, was

* As I find very few of the medical gentlemen have thought it worth their while to tumble over the dirty pages of this publication, I transcribe one passage further, respecting it, from the Edinburgh Review, page 45, that they may be satisfied how little it is worth their while to exhaust either their time or patience on such wretched trash.

"After narrating a nonsensical and despicable story of a patient vaccinated by the Rev. Rowland Hill, who is said to have broken out afterwards into ulcers which were followed by patches of hair, 'some of it like cow's hair!" he breaks out into the following rhapsody of low and miserable buffoonery, which we really believe is unequalled for dullness and vulgarity by any thing that ever issued from Grub Street."

I am satisfied after this, it never can be needed to refer to the passages quoted by the Reviewers, as being fit for a republication by none but Moseley himself.

was never abusive and vulgar, like Dr. Moseley and his antivaccinist adherents, used to make the same calculation on his behalf on the average of one in a hundred. And this he gave as the reason why men of real ability and professional knowledge could scarcely obtain a competent maintainence by their practice, while luxuries of every sort rolled in upon him in the largest abundance he could possibly wish.

While, therefore, the calm dispassionate reasonings of a Jenner, a Moore, a Ring, a Willan, and others, burning now and then with a little anger from some of them, while a scavenger bespatters them with his scoop when dressed in raiment neat and clean. I say, while their arguments go for nothing, the style of Moseley and his adherents proves just the thing; and by the assistance of unexampled effrontery, pompous pretensions to extended erudition, and feigned philanthropy, they are sure to captivate the vulgar mind, and carry all before them; and by these means a wretched plague continues to be kept alive, which by now, as in other countries, might have been almost non-existent throughout the land.

Desirous, therefore, that the wide extended evils of such publications may be counteracted, I presume that you will favourably accept and publish, through the medium of your Journal, some few remarks on the dogmatic falsehoods, or gross miscolourings, that have made their appearance in the last pamphlet of this redoubtable Doctor. I shall only trouble you with such cases as have been personally laid by him to my charge; and if others should have it in their power to contradict what he has narrated respecting their practice also, I am satisfied he may be exposed, as having been the author of such a farrago of inconsistencies and untruths, as perhaps was never exhibited from the press before.

I have already observed how this unfortunate Doctor flounders about in a state of entire uncertainty, whether a disease evidently cutaneous like the measles, small-pox, or though it connects itself with the constitution at large, produces its bad effects almost immediately, and is afterwards soon expelled; or whether it continues to torment the patient to the latest period of life; and then blames me for not inserting a note in one of his former publications, which still more abundantly proves the strange perplexity of his mind, being, as far as I can recollect, for and against the same hypothesis even on the same page. It is marvellous to me, how some people acquire a Doctor's degree.

Now I have in general supposed that all gentlemen of *real* ability in the medical line are decidedly of opinion, that all these cutaneous diseases are only communicated *suo genere*, and that it is through their *effects* and *consequences* alone, from some latent causes in the constitution, that the system is afterwards affected by them.

Dr. Moseley, however, now seems to have it before him as clear as day-light, that this beastly disease, when once implanted, is to branch itself out into a variety of other diseases which never existed before, and which he supposes may continue to torment the patient till the latest period of his life.

It is almost mockery to ask any gentleman of respectability, whether they ever witnessed any of the strange new diseases: The facies bovilla, the scabies bovilla, the tinea bovilla, the elephantiasis bovilla, &c. which he has thought proper to play off upon the credulity of mankind, or whether it is possible they can exist if these cutaneous eruptions communicate no other diseases but themselves.

Now that the cow-pock is still more evidently a cutaneous disease than any other, cannot admit a shadow of a doubt; for, first, even the slightest touch upon the cuticle produces it, nor does the vesicle produced ever transgress the limits designed by Nature for its operation; and a stronger proof still that the cow-pock is more evidently cutaneous than any other disease, appears from this circumstance; in the variolous inoculation, several days elapse before any appearance of disease is produced thereby, while the cow-pock virus produces its almost immediate effect before any constitutional symptoms can possibly exist.

From these considerations I shall take it for granted, that it is the natural conclusion among men of science, that all these cutaneous eruptions are liable to do more or less evil to the constitution, according to the violence of their attack. Even the itch itself, though no real disease, yet let those little tormenting animalcula which create the evil, be permitted to prevail over the surface of the body, such internal constitutional evils will naturally be produced through the system at large as must terminate in death itself.

Now the prejudicial effects of the small-pox, however communicated, are so universally admitted, that to fill page after page by producing a long list of such cases would would be both needless and absurd, tho' at the same time, supposing that the small-pox originated with the camel, what a variety of cases might be produced of camel-faced boys and girls, and camel-haired children, &c. &c. from those who have submitted to the variolous inoculation, supposing that every evil through life of this description was effected by that inoculation. Now what would the fabricators of such idle tales deserve? the scorn and contempt of all that thought it worth their while to read them. As, therefore the cow-pock produces the least possible effect upon the constitution, so, of course, it is scarcely conceivable that the least degree of injury can be communicated to the patient thereby.

Still the cow-pock labours under these difficulties, it is even yet but a new discovery; while some are alarmed because the disease was first communicated from a beast, others are astonished that a disease so slight should prove such a protection against another so contagious and dreadful in its effects. Now, under these considerations, it is no wonder that it was found a very easy business to excite a set of groundless suspicions on the public mind, that many latent evils might secretly lurk under this apparent good. Every eruption therefore, and all glandular tumours that afterwards appear, are imputed to vaccination as the original cause, as though no person upon earth who had once been vaccinated was ever afterwards to suffer from the effects of any scrofulous disease.

It is enough for us that the average of consequences is indisputably in our favour, and to this argument we can with the utmost confidence make our appeal. In my late publication I merely resorted *to matters of fact,* and I still find myself supported by them.

In different parts of England and Wales, I have inoculated with my own hand at least 5,300 subjects; and by instructing others, according to the rules of the Jennerian Institution, I may have promoted, in different parts of the country, the vaccination of perhaps as many more.

Among these I repeatedly make all the inquiry in my power, and as yet, notwithstanding some flying reports, I have not met with a single failure from any quarter whatever.

Now I by no means presume to assert that they never have, nor never will happen, as in the case of variolous inoculation; but so matters at present stand.

To the above list I may add those who were inoculated at the school-room of this chapel, as a station under the

 patronage patronage of the Jennerian Society; that list now amounts to upwards of 4,300 subjects; and here, notwithstanding some slovenly work, till we were under better regulation, for which I am not willing to be answerable, we have been nearly as successful as on the former list. Only one failure, and that of a very slight description, having been produced against us; and on this failure, it may be presently necessary to make some remarks. As to any bad reports respecting the health of those who have been the subjects of vaccination, though so common after the variolous inoculation, they are very rare occurrences indeed; and even beyond all this I can positively assert, I have met with increasing evidence that the cow-pock, instead of implanting these diseases, actually appears to have just the contrary effect. Such was Dr. Jenner's opinion in his first publication on this subject, and all know he is not one of those who was likely to commit himself to public censure by any assertions that were unguarded and rash.

Even the very enemies of vaccination inadvertently give the strongest proof of the truth of this assertion. All know how much the health of the poor is impaired by living in crowded cities and close habitations, where no space can be allowed for cleanliness and a free circulation of air.

Now, out of 4,300 cases and upwards which have been inoculated at this station, and we are just in the situation described before, Dr. Mosely has thought it worth his while to produce *four* against us, who were subjected to scrofulous tumours and diseased constitutions. Alas, for the poor Doctor! after all the diligence exemplified by himself and his *searchers*, after all their public advertisements in order to collect together every evil report from every quarter, that they could not produce at least a list of forty instead of four! two of them however, I believe, are the same I mentioned in a former publication, and now the Doctor has found two more; wonderful discovery! We will for once suppose his stories are precisely correct, and that the vaccine inoculation was the cause of these complaints, and then we will ask the question, Were the same number of subjects to be under the variolous inoculation, what might be expected as the average of bad effects therefrom? What ignorance and prejudice if any were to presume to deny, that it would not be at least ten to one against such rash hands as would wish to encourage and promote that fatal disease.

Thus far I proceed for the present; if this paper be thought thought worthy of your acceptance, it may be followed with another, in which I shall have to expose some other falshoods which have been made public by these antivaccinists, as also present before you some other information which may be well calculated to promote the general good.

*Surry Chapel, Jan.* 15, 1807. ROWLAND HILL.

( To be continued. )

